# Investigation on the Potential Functions of ZmEPF/EPFL Family Members in Response to Abiotic Stress in Maize

**DOI:** 10.3390/ijms25137196

**Published:** 2024-06-29

**Authors:** Rui Liu, Keli Xu, Yu Li, Wanqing Zhao, Hongjing Ji, Xiongbiao Lei, Tian Ma, Juan Ye, Jianhua Zhang, Hewei Du, Shi-Kai Cao

**Affiliations:** 1 School of Life Science, Yangtze University, Jingzhou 434025, China; liuxiaoshuang_6@163.com (R.L.); xukeli2024@163.com (K.X.); tinashecool@163.com (Y.L.); m15062656201@163.com (W.Z.); jjing0105@163.com (H.J.); leixiongbiao627@163.com (X.L.); 19164047603@163.com (T.M.); 18882839330@163.com (J.Y.); 2 Department of Biology, Hong Kong Baptist University, Hong Kong, China; jzhang@hkbu.edu.hk; 3 State Key Laboratory of Agrobiotechnology, The Chinese University of Hong Kong, Shatin, Hong Kong

**Keywords:** abiotic stress, EPF/EPFL, gene family, hormone, bioinformatic analysis, maize

## Abstract

Maize is an important crop used for food, feed, and fuel. Abiotic stress is an important factor affecting maize yield. The *EPF*/*EPFL* gene family encodes class-specific secretory proteins that play an important role in the response to abiotic stress in plants. In order to explore and utilize the EPF/EPFL family in maize, the family members were systematically identified, and their chromosomal localization, physicochemical properties, *cis*-acting element prediction in promoters, phylogenetic tree construction, and expression pattern analysis were carried out using bioinformatics techniques. A total of 18 ZmEPF/EPFL proteins were identified in maize, which are mostly alkaline and a small portion acidic. Subcellular localization results showed that ZmEPF6, ZmEPF12, and ZmEPFL2 are localized in the nucleus and cytoplasm. Analysis of *cis*-acting elements revealed that members of the ZmEPF/EPFL family contain regulatory elements such as light response, anoxic, low temperature, and hormone response regulatory elements. RT-qPCR results showed that these family members are indeed responding to cold stress and hormone treatments. These results of this study provide a theoretical basis for improving the abiotic stress resistance of maize in future research.

## 1. Introduction

Agricultural ecosystems have been adversely affected by abiotic stress, such as high temperature, low temperature, drought, salt, and so on [1]. Stomata is one of the key factors for plants to resist adversity stresses, whose size and density change with the environment [2]. By altering stomatal density, pore size, and switching time, carbon dioxide uptake in plant photosynthesis can be optimized, and external abiotic stresses can be resisted [3]. A large number of ROS accumulated in guard cells promote stomatal closure through activation of calcium channels in the plasma membrane in response to a variety of abiotic stresses [4]. Plants could increase drought tolerance by regulating stomatal closure and reducing water transpiration [5,6].

The fossil record indicates that terrestrial plants had stomatal-like structures on their surface over 400 million years ago [7]. This feature emerged independently in newly evolved terrestrial plant strains [8]. Stomata are composed of guard cells and the pores formed around them, which exist on the surface of most terrestrial plants and act as microscopic pressure-driven valves [7]. Each stoma is surrounded by two highly specialized cells called Guard Cells, which require stereotypical asymmetric and symmetric cell division [9]. Its cell development process strictly follows the cell division program, ensuring that there is at least one interval of cell separation between each mature stoma [10].

In order to facilitate the correct development of stomata and the absence of intercellular separation between stomata, some extracellular and plasma membrane-bound proteins are essential for the coordinated development of stomata and signaling between Epidermal Pavement Cells [11]. These signals include EPF (EPIDERMAL PATTERNING FACTOR) and EPFL (EPIDERMAL PATTERNING FACTOR-Like) signal peptides, leucine-rich repetitive ER (ERECTA) series membrane receptor kinases ERECTA/ERECTA-LIKE1 (ERL1), and Leu-rich repetitive membrane protein TMM (Too Many Mouths) [10]. During stomatal development, ER family proteins regulate the direction of differentiation of MMC (Meristemoid Mother Cells) and are regulated by EPF [12].

So far, members of the *EPF*/*EPFL* family have been identified in several species, such as moss (*Physcomitrium patens*), barley (*Hordeum vulgare*), rice (*Oryza sativa*), wheat (*Triticum aestivum* L.), and *Arabidopsis thaliana* [13,14,15,16,17]. For example, moss has conserved homologs of EPF, which function together to facilitate the correct development of stomata [13]. Plants develop stomata in a manner that employs multiple transcription factors and EPF ligands to more precisely control subsequent differentiation [18]. In barley, overexpression of HvEPF1 restricts the access of HvEPF1 to the stomatal developmental pathway and significantly enhances water use efficiency, drought tolerance, and soil water retention performance [14]. In rice, overexpression of OsEPF1 and OsEPF2 could reduce the stomatal density and stomatal conductance in leaves, which led to improved water use efficiency [15,19]. Moreover, several studies have demonstrated that EPF/EPFL family members are involved in the stomatal development process in *Arabidopsis thaliana*. *EPF1* encodes a small secretory protein that is expressed in stomatal precursor cells and controls stomatal development by regulating asymmetric cell division in the MMC [10]. *EPF2* regulates the stomatal density of mature leaves and controls the number of cells entering the stomata during leaf development [20]. Elevated expression of EPFL9 increases stomatal density and aggregation [21]. However, the control of stomatal aggregation by EPFL9 and EPF1, as well as the regulation of stomatal density by EPFL9 and EPF2, are independent of each other [22].

Besides involvement in stomatal regulation, EPFL members are also involved in plant growth, fertility, and yield regulation. For instance, OsEPFL1 has the function of elongating awns in rice [23]. OsEPFL2 is involved in the development of awns and large grains, regulating grain length and grain weight [24]. Furthermore, OsEPFL6, OsEPFL7, OsEPFL8, and OsEPFL9 negatively regulate the number of spikelets per spike. The *osepfl6 osepfl7 osepfl9* triple mutants significantly increased grain yield without affecting spikelet fertility, and specific inhibition of the OsEPFL6-OsER1, OsEPFL7-OsER1, and OsEPFL9-OsER1 ligand receptors could optimize rice panicle architecture [25].

Although the number of EPF/EPFL homologs has been identified in several species, there is still a lack of systematic identification and related research reports on the members of the EPF/EPFL family in maize. In this paper, we conducted a series of bioinformatics analyses to systematically identify and analyze the members of the *EPF/EPFL* gene family in maize. These results contribute to a better understanding of the evolutionary history and functional mechanisms of the EPF/EPFL family in maize.

## 2. Results

### 2.1. Gene Structure Analysis and Protein-Conserved Motif and Domain Prediction

**Figure 1:**
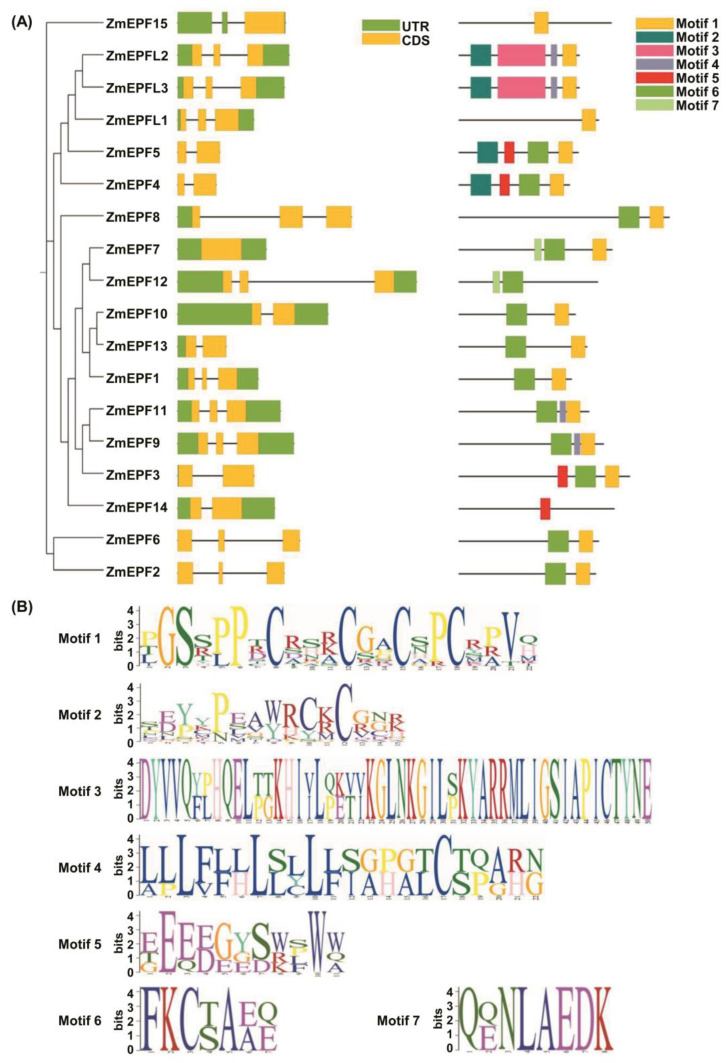
Gene structure and conserved motif prediction of *ZmEPF*/*EPFL* family members. (**A**) The phylogenetic relationship, gene structure, and conserved motifs of cDNA sequences are listed on the picture’s left, middle, and right sides, respectively. Gray lines represent introns, green squares represent UTR (untranslated region), and yellow squares represent exons (middle). Gray lines represent proteins expressed by family members; green, red, gray, and yellow boxes represent different motifs (right). (**B**) The conserved sequences and main protein domains.

To identify the EPF/EPFL family members in maize, we downloaded the maize genome DNA, cDNA, protein sequence, and maize annotation files from the Ensembl Plants website and performed the protein blast using the Arabidopsis EPF/EPFL family members. A total of 18 EPF/EPFL members were identified in maize, named ZmEPF1-15 and ZmEPFL1-L3, respectively (Figure 1). To visualize the gene structure of maize EPF/EPFL family members, we used TBtools-II (V2.042) software to draw a schematic diagram of gene structure. All 17 maize *EPF*/*EPFL* genes contain one or two introns, except for *ZmEPF7,* which is an intronless gene (Figure 1A).

Next, a prediction of the protein-conserved domain and motif of all maize EPF/EPFL family members was performed. Results of conserved motif prediction showed that there are seven motifs in the maize EPF/EPFL family members, from motif 1 to 7 (Figure 1B). Three family members have one motif, seven members contain two motifs, three members have three motifs, and four members have four motifs (Figure 1A). Prediction of domain showed that there are three conserved domains in the maize EPF/EPFL family members, including the EPF domain, the stomagen-like superfamily domain, and the PLN03207 domain (Figure S1). Among the maize EPF/EPFL family members, 12 members contain EPF domains. ZmEPFL1 contains a stomagen-like superfamily domain, and ZmEPFL2 and L3 contain PLN03207 domains (Figure S1).

### 2.2. Phylogenetic Relationship Analysis

**Figure 2:**
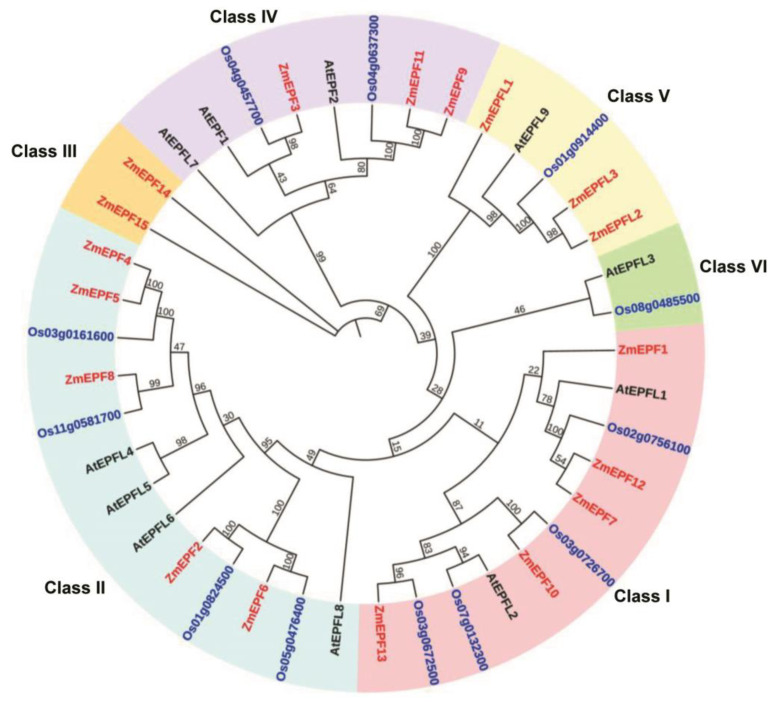
Phylogenetic tree of EPFs and EPFLs in *Zea mays*, *Oryza sativa*, and *Arabidopsis thaliana*. Red font indicates EPF/EPFL family members in *Zea mays*, black font indicates EPF/EPFL family members in *Arabidopsis thaliana*, and blue font indicates EPF/EPFL family members in *Oryza sativa*, and different color backgrounds highlight different classes: pink background highlights Class I, blue background highlights Class II, yellow background highlights Class III, purple background highlights Class IV, light yellow background highlights Class V, and green background highlights Class VI.

To analyze the phylogenetic relationships of the ZmEPF/EPFL family, phylogenetic tree construction of EPF/EPFL proteins was completed in the three species of *Arabidopsis thaliana*, *Oryza sativa*, and *Zea mays* (including 11 Arabidopsis proteins, 12 rice proteins, and 18 maize proteins) using the MEGA 7 (V7.0.26) software (Figure 2). Six subfamilies were classified, from Class I to Class VI subfamilies. There are five members in the Class I subfamily, five in Class II, two in Class III, three in Class IV, three in Class V, and zero in Class VI, respectively (Figure 2).

In addition, we performed the collinearity analysis of EPFs and EPFLs in different species (*Arabidopsis thaliana*, *Zea mays*, and *Oryza sativa*) (Figures S2 and S3). Results showed that there are three homologous genes between *Arabidopsis thaliana* and *Zea mays*, while there are 12 homologous genes between *Zea mays* and *Oryza sativa* (Figure S2). In *Zea mays*, there are five homologous pairs of *EPF*/*EPFL* genes, including *ZmEPF2* and *6*, *ZmEPF4* and *5*, *ZmEPF7* and *12*, *ZmEPF9* and *11*, *ZmEPFL2* and *L3* (Figure S3). These results suggested that maize EPF/EPFL family members are more closely related to rice compared with Arabidopsis, consisting of the phylogenetic tree.

### 2.3. Physicochemical Property Prediction

To get information on the physicochemical properties of ZmEPF/EPFL family members, a comprehensive analysis of the ZmEPF/EPFL family was performed, including subcellular localization, protein length, molecular mass, theoretical isoelectric point (pI), instability index, and grand average of hydropathicity (GRAVY) (Table 1). As shown in Table 1, the relative protein length of the ZmEPF/EPFL members ranges from 113 to 216 amino acids, the relative molecular weight is from 12,115.97 to 23,070.52 Da, and the pI is between 6.81 and 10.7. Only one family member has an isoelectric point less than 7, and the rest have isoelectric points greater than 7. The instability indexes (II) were computed to range from 46.20 to 76.84, indicating that all family members may be unstable. The GRAVY ranges from −0.032 to −0.680, indicating that all the family members may be hydrophilic proteins.

**Figure 3:**
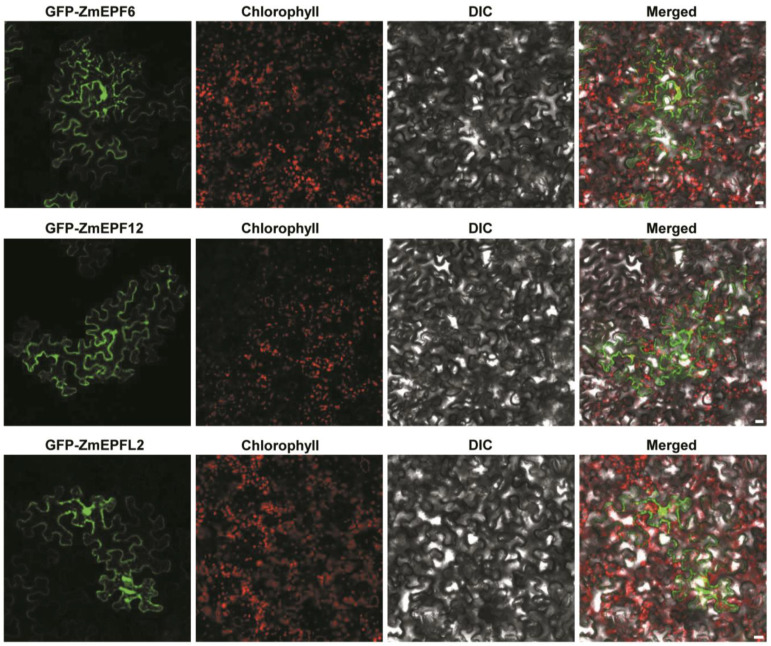
The subcellular localization of ZmEPF6, ZmEPF12, and ZmEPFL2 in *N. benthamiana*. ZmEPF6, ZmEPF12, and ZmEPFL2 fused with N-terminal GFP were infiltrated in *N. benthamiana*, respectively. Scale bar, 20 µm.

Subcellular localization prediction revealed that ZmEPF/EPFL family members may localize in the nucleus, plasma membrane, chloroplast, or cytoplasm (Table 1). To further study the characteristics of these proteins, we fused them with pGWB6-ZmEPF6, ZmEPF12, and ZmEPFL2, transformed tobacco leaf epidermis through Agrobacterium-mediated methods, and observed fluorescence signals using fluorescence confocal microscopy, respectively. Results showed that the green fluorescence signals of pGWB6-ZmEPF6, 12, and L2 were observed in the nucleus, cytoplasm, cell wall, or cell membrane (Figure 3), partially consistent with the results of subcellular localization prediction (ZmEPF6: chloroplast, nucleus, and cytoplasm; ZmEPF12: nucleus; ZmEPFL2: chloroplast, nucleus, and cytoplasm). Regarding whether ZmEPF6 and L2 have chloroplast localization signals, this needs to be further investigated by constructing vectors in which GFP is fused to the C-terminus of the target proteins.

### 2.4. Identification and Chromosome Distribution

**Figure 4:**
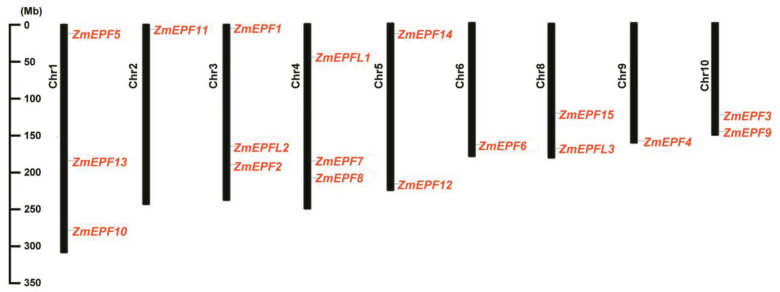
Gene distribution of ZmEPF/EPFL family members in chromosomes. The black bars represent the chromosomes, the gray lines indicate the location of the genes on the chromosomes, and the length of the chromosomes is shown on the left scale.

Based on genome annotation information, we drew the chromosome distribution map of 18 maize EPF/EPFL family members using the TBtools-II software. Results showed that all 18 *ZmEPF*/*EPFL* members were distributed on nine chromosomes randomly from chromosome 1 to 10, except for 7 (Figure 4).

### 2.5. Analysis of cis-Acting Elements in Promoters

**Figure 5:**
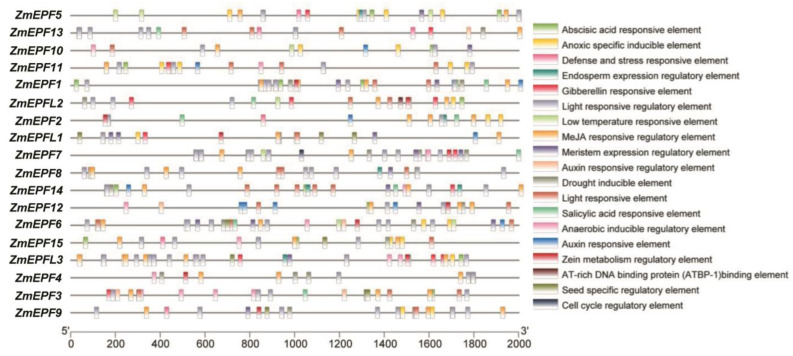
*Cis*-element analysis in promoters of ZmEPF/EPFL family members. Gray lines represent promoters corresponding to family members, various colored boxes represent different *cis*-elements, the *cis*-element corresponding to each colored box is shown on the right side of the diagram, and promoter lengths are shown on the scale below.

In order to understand which factors may be related to ZmEPF/EPFL family members, we performed a *cis*-acting element analysis of the promoter region of ZmEPF/EPFL family members. Results showed that the promoters of the family members contain nineteen *cis*-element (Figure 5), including abscisic acid responsive element (ten members), anoxic specific inducible element (ten members), defense and stress responsive element (six members), endosperm expression regulatory element (five members), gibberellin responsive element (nine members), light responsive regulatory element (eighteen members), low temperature responsive element (five members), MeJA responsive regulatory element (eighteen members), meristem expression regulatory element (eleven members), auxin responsive regulatory element (three members), drought inducible element (seven members), Light responsive element (thirteen members), salicylic acid responsive element (eight members), anaerobic inducible regulatory element (nine members), auxin responsive element (eight members), zein metabolism regulatory element (eight members), AT-rich DNA binding protein binding element (one member), seed specific regulatory element (four members), and cell cycle regulatory element (one member).

### 2.6. Expression Pattern Analysis

**Figure 6:**
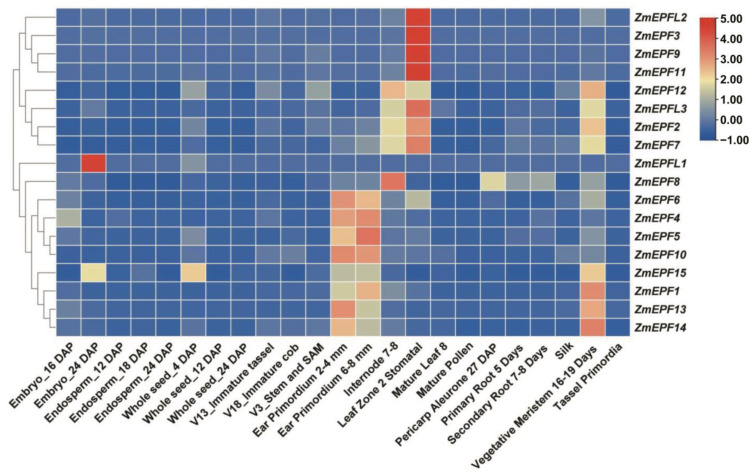
Expression pattern of *ZmEPF/EPFL* family members in different tissues. The horizontal coordinates represent the tissues from which the material was taken, and the different colors indicate the transcript levels of the genes of each member of the family.

To investigate the expression pattern of ZmEPF/EPFL family members, a spatiotemporal expression heatmap was established based on the transcriptome data (Figure 6). As shown in Figure 6, *ZmEPFL1* exhibits high expression levels in embryos (24 DAP), *ZmEPF3*, *9*, *11*, and *L2* exhibit high expression level in leaf zone 2 (stomatal) comparing with other members. *ZmEPF2*, *7*, *12*, and *L3* exhibit relatively moderate expression levels in internode 7–8, leaf zone 2 (stomatal), and vegetative meristem (16–19 days). ZmEPF15 exhibits a medium expression level in embryos (twenty-four DAP) and whole seeds (four DAP).

### 2.7. Expression Levels of ZmEPF/EPFL Genes in Response to Abiotic Stresses

**Figure 7:**
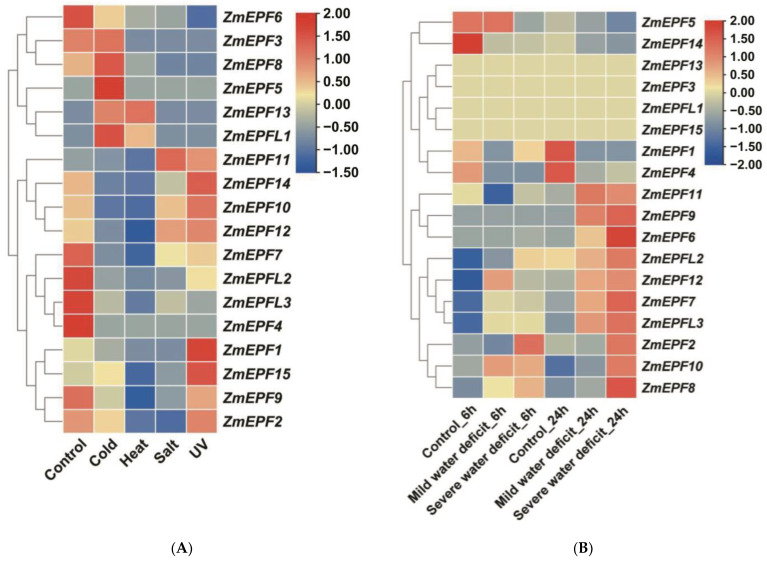
Expression pattern of *ZmEPF*/*EPFL* family members under different stresses. (**A**) Expression pattern of *ZmEPFs*/*EPFLs* under cold, heat, salt, or UV stress. (**B**) Expression pattern of *ZmEPFs*/*EPFLs* under water deficit stress.

To investigate the potential response to abiotic stresses of ZmEPF/EPFL family members, expression heatmaps under different abiotic stresses were established, including cold stress, heat stress, salt stress, UV stress, and water deficit stress (Figure 7). Results of heatmaps showed that expression of six members increased under cold stress, including *ZmEPF3*, *5*, *8*, *13*, *15*, and *L1*. The expression of two members (*ZmEPF13* and *L1*) increased under heat stress. Under salt stress, only two members (*ZmEPF11* and *12*) showed increased expression. Under UV stress, seven members exhibited increased expression levels, including *ZmEPF1*, *2*, *10*, *11*, *12*, *14*, and *15* (Figure 7A).

Furthemore, the transcription level of ZmEPF/EPFL family members was also analyzed under different degrees of water deficit (Figure 7B). There were no significant differences in the expression of the four family members in the presence or absence of a water deficit, including *ZmEPF3*, *13*, *15*, and *L1*. After 6 h under mild water deficit stress, the transcription levels of five members increased, including *ZmEPF7*, *8*, *10*, *12*, and *L3*. After 24 h under mild water deficit stress, the transcription levels of seven members increased, including *ZmEPF6*, *7*, *9*, *11*, *12*, *L2*, and *L3*. Compared with a mild water deficit, more family members responded to a severe water deficit. After 6 h under severe water deficit stress, the transcription levels of seven members increased, including *ZmEPF2*, *7*, *8*, *10*, *12*, *L2*, and *L3*. After 24 h under severe water deficit stress, the transcription levels of ten members increased, including *ZmEPF2*, *6*, *7*, *8*, *9*, *10*, *11*, *12*, *L2*, and *L3*.

**Figure 8:**
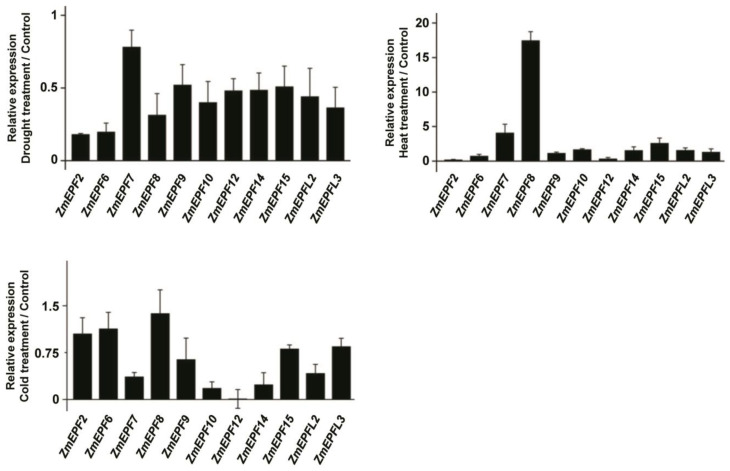
Expression level of *ZmEPF*/*EPFL* members under drought, heat, and cold treatments. Expression levels of *ZmEPF2*, *6*, 7, *8*, *9*, *10*, *12*, *14*, *15*, *L2*, and *L3* were detected by RT-qPCR under drought, heat, and cold treatments. Data are means (±SE) of three biological replicates.

We detected the expression levels of 11 *ZmEPF*/*EPFL* family members under drought, heat, and cold treatments by RT-qPCR, including *ZmEPF2*, *6*, *7*, *8*, *9*, *10*, *12*, *14*, *15*, *L2*, and *L3* (Figure 8). Results showed that the expression of all these genes was reduced under drought stress. The expression of *ZmEPF7*, *8*, and *15* was induced under heat stress, and *ZmEPF7*, *10*, *12*, *14*, and *L2* were significantly reduced under cold stress (Figure 8). These results suggested that *ZmEPF*/*EPFL* members differentially respond to drought, heat, and cold treatments.

**Figure 9:**
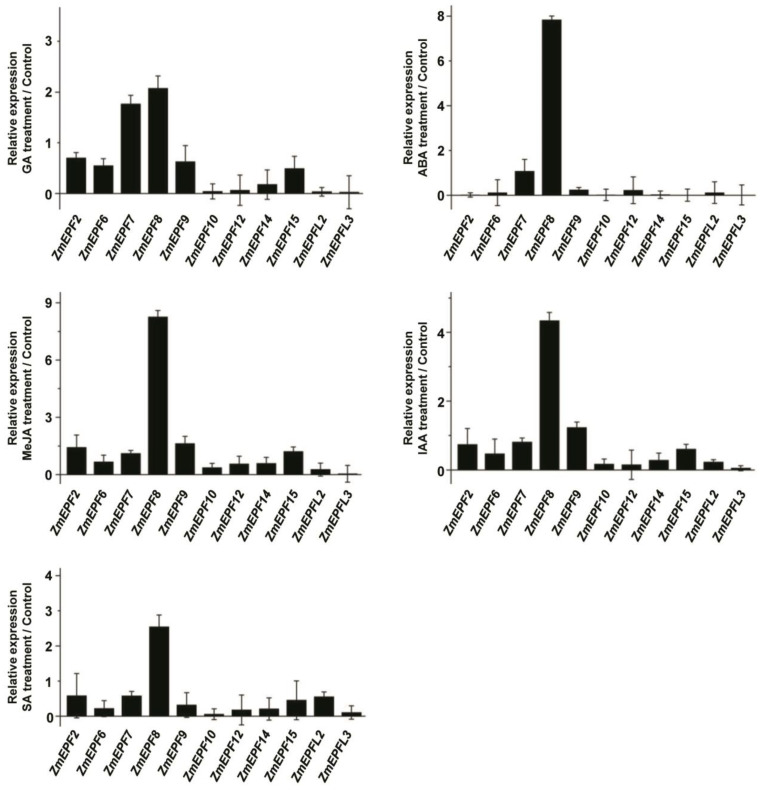
Expression level of *ZmEPF*/*EPFL* members under different hormone treatments. Expression levels of *ZmEPF2*, *6*, 7, *8*, *9*, *10*, *12*, *14*, *15*, *L2*, and *L3* were detected by RT-qPCR under different hormone treatments including ABA, GA, IAA, MeJA, and SA treatments. Data are means (±SE) of three biological replicates.

In addition, the expression levels of 11 *ZmEPF*/*EPFL* family members under different hormone treatments were detected by RT-qPCR (Figure 9), including *ZmEPF2*, *6*, *7*, *8*, *9*, *10*, *12*, *14*, *15*, *L2*, and *L3*. Results showed that the expression of *ZmEPF6*, *9*, *10*, *14*, *and L3* was significantly reduced, while *ZmEPF8* was induced under SA treatment. Under GA treatment, expression of *ZmEPF10*, *12*, *14*, *L2*, and *L3* was reduced, while *ZmEPF8* was induced. Under ABA treatment, except for *ZmEPF7*, the expression of *ZmEPF8* was obviously induced by ABA, while the expression levels of other members were reduced significantly. Under JA treatment, the expression of *ZmEPF10*, *L2*, and *L3* was significantly reduced, while *ZmEPF8* was induced obviously. Under IAA treatment, the expression of *ZmEPF6*, *10*, *14*, *L2*, and *L3* was significantly reduced, while *ZmEPF8* was induced obviously. These results suggested that *ZmEPF*/*EPFL* members respond to different hormone treatments.

## 3. Discussion

Previous studies reported that EPF/EPFL proteins participate in various biological processes in several plant species, including the regulation of stomatal density and aggregation, plant growth, fertility, and yield. However, there is still a lack of systematic identification and comprehensive analyses of the ZmEPF/EPFL family in evolution. In this work, we performed bioinformatics analyses of the EPF/EPFL family members in maize. A total of 18 ZmEPF/EPFL proteins were identified. Their phylogenetic analysis, physicochemical properties, *cis*-acting element prediction in promoters, and expression pattern analysis were carried out. These results contribute to a better understanding of the evolutionary history and functional mechanisms of the EPF/EPFL family in maize.

Phylogenetic tree analysis showed some EPF/EPFL members from monocotyledons (rice and maize) and dicotyledons (Arabidopsis) were distributed in the same branch (Figure 2). For example, ZmEPF3 clusters with AtEPF1 and Os04g0457700 (OsEPF2) and ZmEPF9 and ZmEPF11 clusters with AtEPF2 and Os04g0637300 (OsEPF1), all belonging to Class IV (Figure 2), suggest that the EPF/EPFL family emerged before the differentiation of dicotyledons and monocotyledons. EPF/EPFL members were divided into six subgroups (Figure 2), suggesting that they were highly conserved during the evolution of Arabidopsis, rice, and maize. Moreover, AtEPF1 and AtEPF2 act as negative regulatory factors to regulate stomatal development [10,16], *OsEPF1* and *OsEPF2* regulate stomatal density [19], and overexpression of OsEPF1 or OsEPF2 significantly reduces the frequency of stomatal density in Arabidopsis *epf2* and *epf1epf2* mutants, demonstrating that EPF1 and EPF2 are indeed functionally conserved between rice and Arabidopsis. This evidence implies that ZmEPF3, 9, and 11 may function in stomatal development and/or density regulation in maize due to their co-cluster with OsEPF1 and OsEPF2 in Class IV. Similarly, ZmEPFL1, L2, and L3 clustered in Class V with AtEPFL9 (Figure 2), and AtEPFL9 functions as a positive regulatory factor in regulating stomatal density and degree of stomatal aggregation [15,21], suggesting that ZmEPFL1, L2, and L3 also may function in stomatal development in maize. These results indicated that some EPF/EPFL homologies were conserved during evolution.

It was previously reported that the EPF/EPFL gene can appropriately reduce stomatal density in the early stages of plant development, reducing transpiration and water loss while maintaining photosynthesis, thereby improving plant drought resistance and water use efficiency [26], suggesting that the *EPF*/*EPFL* gene family may play an important role in defense. *Cis*-acting element analysis showed that ZmEPF/EPFL members contained defense and stress-responsive elements, low temperature-responsive elements, and drought-inducible elements (Figure 5), speculating that ZmEPF/EPFL members may respond to stress signals. For example, the ZmEPF11 promoter region contains a defense and stress-responsive element (Figure 5), and it is expressed and induced under salt and water deficit, suggesting that it may play a role in response to flooding, salt, UV, and drought (Figure 7). The ZmEPF9 promoter region contains a number of elements, including a defense and stress-responsive element and a drought-inducible element (Figure 5), and it is expressed as induced under water deficit (Figure 7B), suggesting that it may play a role in response to waterlogging and drought. Additionally, RT-qPCR results showed that the expression of *ZmEPF7*, *8*, and *15* was induced under heat stress and *ZmEPF7*, *10*, *12*, *14*, and *L2* were reduced under cold stress (Figure 8). These data indicated that *ZmEPF*/*EPFL* members respond to abiotic stresses.

Plant hormones play an important role in the formation and development of stomata [27,28,29,30]. For example, auxin (IAA) regulates stomatal development in guard cells. The higher the concentration of auxin, the greater the degree of stomatal opening [31]. Several EPF/EPFL members were reported to function in stomatal development and regulation [10,16,21]. How these members and hormone signals function in the regulation of stomata together is unknown in maize. *Cis*-acting element analysis of the promoter region of ZmEPF/EPFL was performed (Figure 5). Results showed that *ZmEPF3*, *12*, and *13* contain auxin-responsive regulatory elements, speculating that these three members may be involved in stomatal development through responding to auxin signals. All ZmEPF/EPFL members contained methyl jasmonate-responsive elements (Figure 5), which is consistent with previous studies on the inhibition of stomatal development by low concentrations of jasmine [32]. Additionally, RT-qPCR results showed that the expression of some *ZmEPF/EPFL* was inhibited or induced under hormone treatments (Figure 9). These data implied that ZmEPF/EPFL members may participate in stomatal development by responding to hormone signals.

In this study, we identified maize EPF/EPFL family members and their potential functions in stomatal development and stress responses. As to what the specific biological functions of each family member are, they need to be analyzed through further in-depth studies. The above exploration results provide a theoretical basis for facilitating further research on the function of *ZmEPF*/*EPFL* genes.

## 4. Materials and Methods

### 4.1. Materials

All materials used in the experiment were sourced from the maize B73 inbred line preserved in the laboratory. Plump seeds were selected to be planted in a tissue culture room in a paper cup filled with nutrient soil. The samples were harvested at the three leaf and one heart stages, frozen in liquid nitrogen, and then stored in a −70 °C refrigerator for subsequent experiments.

Seedlings at the three leaves and one heart stage were treated with low temperature (placed in a transparent refrigerator at 4 °C) for 24 h, methyl jasmonate (MeJA, 0.1 mmol/L), abscisic acid (ABA, 0.1 mmol/L), salicylic acid (SA, 0.1 mmol/L), gibberellin (GA, 0.1 mg/L), and auxin (IAA, 0.1 mg/L) for 6 h, respectively. Mock treatment was used as the corresponding control (CK), with three biological replicates for each treatment. Samples from each treatment were harvested for subsequent RT-qPCR analysis.

### 4.2. Identification of ZmEPF/EPFL Family Members and Analysis of Protein Physicochemical Properties

The protein sequences of 11 Arabidopsis EPF/EPFL family members were obtained from the TAIR website. Maize genome sequences, proteome sequences, and annotation files were downloaded from the ensembl plant (https://plants.ensembl.org/index.html (accessed on 7 January 2024)) database. The proteins containing the same domain were selected via protein bidirectional BLAST using TBtools-II software, and the maize EPF/EPFL family members were verified by EPF (Pfam17181), stomagen-like superfamily, and PLN03207 domain [33,34]. The protein physicochemical properties of maize EPF/EPFL family members were analyzed online using ExPASy (https://www.expasy.org/ (accessed on 7 January 2024)) website, including relative molecular mass, theoretical isoelectric point, instability index, and the grand average of hydropathicity [35]. Subcellular localizations of ZmEPF/EPFL family members were predicted using Cell-Ploc (http://www.csbio.sjtu.edu.cn/bioinf/Cell-PLoc-2/ (accessed on 8 January 2024)) combined with CELLO (http://cello.life.nctu.edu.tw/ (accessed on 8 January 2024)) online website.

### 4.3. Distribution of ZmEPF/EPFL Family Members on the Chromosome

Based on the GTF file, the chromosome location information was obtained from gene IDs of the identified maize EPF/EPFL family members. Distribution map on chromosomes of maize EPF/EPFL family members using TBtools-II software [33,34].

### 4.4. Gene Structure Analysis of ZmEPF/EPFL Family Members

The genomic DNA sequences of maize EPF/EPFL family members were obtained by NCBI (https://www.ncbi.nlm.nih.gov/ (accessed on 1 January 2024)) and Maize GDB (http://www.maizegdb.org/ (accessed on 1 January 2024)), and the gene structure maps were drawn using GSDS (http://gsds.cbi.pku.edu.cn/ (accessed on 9 January 2024)) online software.

### 4.5. Conserved Motif, Domain Prediction, and Phylogenetic Tree Construction of ZmEPF/EPFL Proteins

Conserved motifs of maize EPF/EPFL proteins were predicted using the online MEME (http://meme-suite.org/tools/meme (accessed on 9 January 2024)) software. Conserved domains of maize EPF/EPFL proteins were predicted using CD search tool on the NCBI official website. The conserved motif and domain of maize EPF/EPFL proteins were visualized using the Gene Structure View (Advanced) function of TBtools-II software. All the EPF/EPFL protein sequences of maize, rice, and Arabidopsis were aligned via ClustalW method, and the phylogenetic tree was constructed by MEGA7 (V7.0.26) [36]. The phylogenetic tree was further beautified using the iTol online website (https://itol.embl.de/ (accessed on 25 January 2024)).

### 4.6. Analysis of cis-Acting Elements in the Promoters of ZmEPF/EPFL Family Members

The upstream 2000 bp promoter sequences of the ZmEPF/EPFL transcription start site were obtained according to the official maize database MaizeGDB (https://maizegdb.org/ (accessed on 18 January 2024)). The *cis*-acting elements were predicted using the online tool PlantCARE (https://bioinformatics.psb.ugent.be/webtools/plantcare/html/ (accessed on 12 February 2024)), and figures of *cis*-acting elements were drawn using TBtools-II software [33,34].

### 4.7. Expression Patterns of ZmEPF/EPFL Family Members

The transcriptome data of maize EPF/EPFL family members in multiple tissues and under abiotic stresses were downloaded from qTeller (https://qteller.maizegdb.org/genes_by_name_B73v5.php (accessed on 27 February 2024)). The heatmaps were drawn using TBtools-II software [33,34].

### 4.8. Collinearity Analysis

The genome files and gene annotation files of *Zea mays*, *Oryza sativa*, and *Arabidopsis thaliana* were obtained from the Ensembl Plants database, and the intra-species covariate analysis of *Zea mays* and inter-species covariate analysis of *Zea mays*, *Oryza sativa*, and *Arabidopsis thaliana* were plotted by the MCScanX function in the TBtools-II software [33,34].

### 4.9. RNA Extraction and Real-Time Fluorescence Quantitative PCR

The total RNAs were extracted from the leaves of each treated plant using an RNA-easy isolation reagent (Vazyme Biotech Co., Ltd., Nanjing, China). The first strands of cDNA were synthesized using the HiFiScript cDNA Synthesis Kit (Vazyme Biotech Co., Ltd.) and then used as the templates of the real-time fluorescence quantitative PCR (qPCR) (Vazyme Biotech Co., Ltd.) for further hormone treatments and cold stress-responsive analysis. The RT-qPCR was performed on the CFX96 real-time PCR instrument (Bio-Rad, Hercules, CA, USA), and *ZmActin* (*GRMZM2G126010*) was used as the reference gene. All the primers are listed in Supplementary Table S1. Three biological and technical replicates are performed, respectively.

### 4.10. Subcellular Localization

For subcellular localization, the full-length ZmEPF6, ZmEPF12, and ZmEPFL2 were cloned into binary vector pGWB6, respectively. Agrobacterium tumefaciens strain (*GV3101*) harboring GFP-ZmEPF6, GFP-ZmEPF12, and GFP-ZmEPFL2 constructs were transiently expressed in tobacco epidermal cells, as previously described [37]. The GFP signals were observed and imaged using ZEISS LSM 880 confocal microscope (Carl-Zeiss, Jena, Germany).

## Figures and Tables

**Table 1:** Prediction of physicochemical properties of the maize EPF/EPFL family members.

Gene ID	Protein Name	Subcellular Localization	Protein Length (aa)	Molecular Mass (Da)	Theoretical pI	Instability Index	Grand Average of Hydropathicity
Zm00001eb121050	ZmEPF1	Nucleus	115	12,115.97	9.30	54.00	−0.203
Zm00001eb149400	ZmEPF2	Plasma membrane, Nucleus	140	15,319.77	9.44	71.03	−0.309
Zm00001eb423950	ZmEPF3	Nucleus	175	18,452.86	7.57	61.90	−0.069
Zm00001eb403370	ZmEPF4	Nucleus	113	12,325.37	9.91	69.28	−0.174
Zm00001eb004650	ZmEPF5	Plasma membrane, Nucleus	122	13,264.23	9.39	75.99	−0.313
Zm00001eb290530	ZmEPF6	Chloroplast, Nucleus, Cytoplasm	143	14,827.99	9.49	54.09	−0.031
Zm00001eb194040	ZmEPF7	Chloroplast, Nucleus	157	16,999.45	9.13	55.22	−0.265
Zm00001eb200540	ZmEPF8	Chloroplast, Nucleus	216	23,070.52	10.7	76.84	−0.278
Zm00001eb431780	ZmEPF9	Plasma membrane	148	15,291.78	8.07	46.20	0.194
Zm00001eb055900	ZmEPF10	Nucleus	119	13,069.02	9.78	63.50	−0.680
Zm00001eb069150	ZmEPF11	Plasma membrane	133	13,454.54	8.43	52.04	0.180
Zm00001eb255380	ZmEPF12	Nucleus	143	15,175.36	9.04	56.26	−0.267
Zm00001eb032920	ZmEPF13	Chloroplast	131	13,665.28	9.98	59.87	−0.582
Zm00001eb217540	ZmEPF14	Nucleus	159	17,542.06	8.36	55.92	−0.233
Zm00001eb352020	ZmEPF15	Nucleus, Cytoplasm	156	16,344.11	10.00	52.98	−0.724
Zm00001eb174960	ZmEPFL1	Plasma membrane	143	15,131.95	6.81	61.21	−0.376
Zm00001eb143570	ZmEPFL2	Chloroplast, Nucleus, Cytoplasm	123	13,623.8	8.83	47.80	−0.062
Zm00001eb364920	ZmEPFL3	Plasma membrane	123	13,615.88	8.92	52.74	−0.032

## Data Availability

Data are contained within the article and supplementary materials.

## Supplementary Materials

The following supporting information can be downloaded at: https://www.mdpi.com/article/10.3390/ijms25137196/s1.
