# Supplementary material for: Investigation on the Potential Functions of ZmEPF/EPFL Family Members in Response to Abiotic Stress in Maize
Source: Int J Mol Sci. 2024 Jun 29;25(13):7196. doi: 10.3390/ijms25137196 (PMC11241529; doi:10.3390/ijms25137196)
Supplement: Supplementary file 1 [file ijms-25-07196-s001.zip › Supplementary figures and table/Supplementary figures.pptx]

## Slide 1
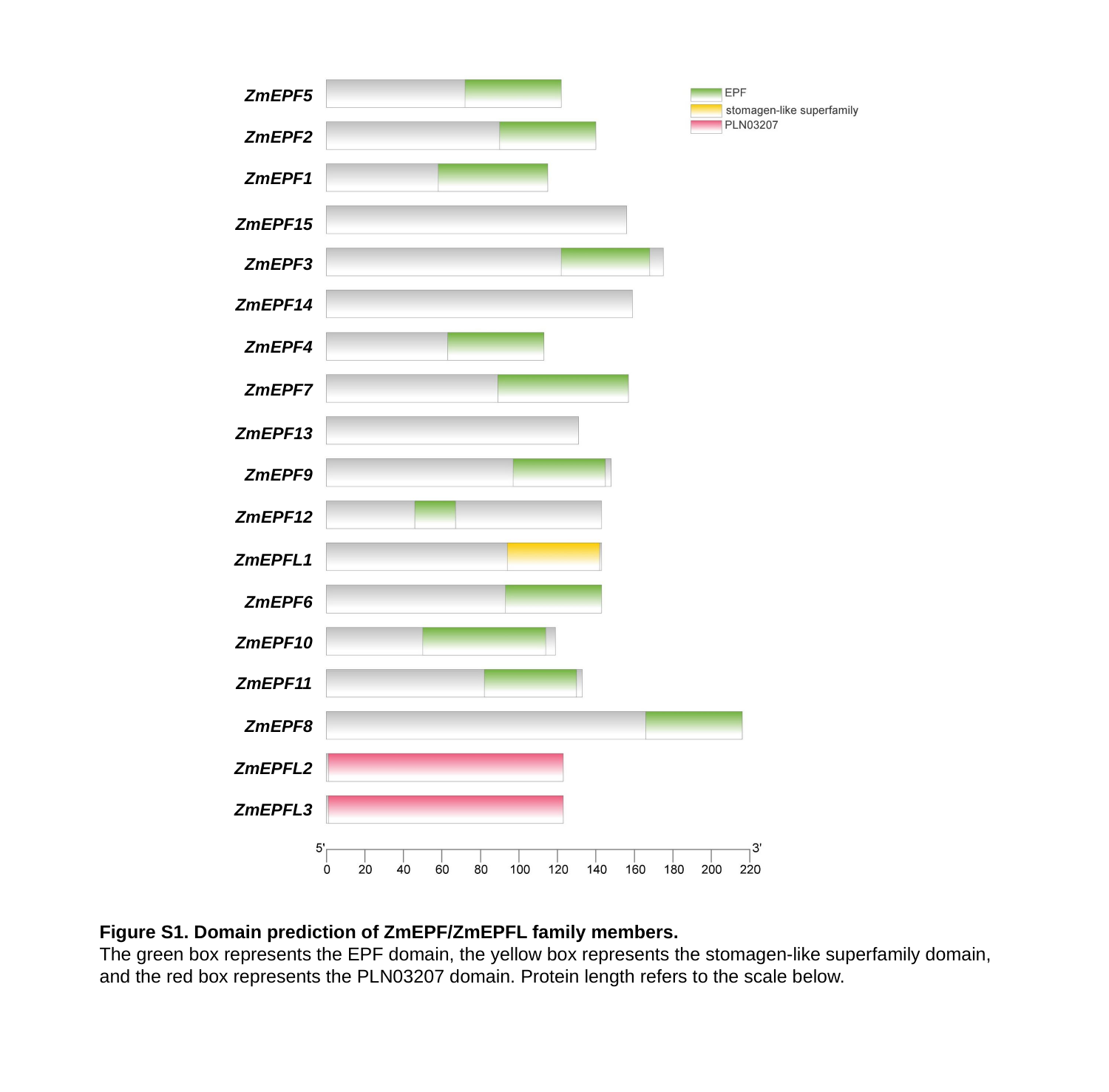

ZmEPF5
ZmEPF2
ZmEPF1
ZmEPF15
ZmEPF3
ZmEPF14
ZmEPF4
ZmEPF7
ZmEPF13
ZmEPF9
ZmEPF12
ZmEPFL1
ZmEPF6
ZmEPF10
ZmEPF11
ZmEPF8
ZmEPFL2
ZmEPFL3
Figure S1. Domain prediction of ZmEPF/ZmEPFL family members.
The green box represents the EPF domain, the yellow box represents the stomagen-like superfamily domain, and the red box represents the PLN03207 domain. Protein length refers to the scale below.

## Slide 2
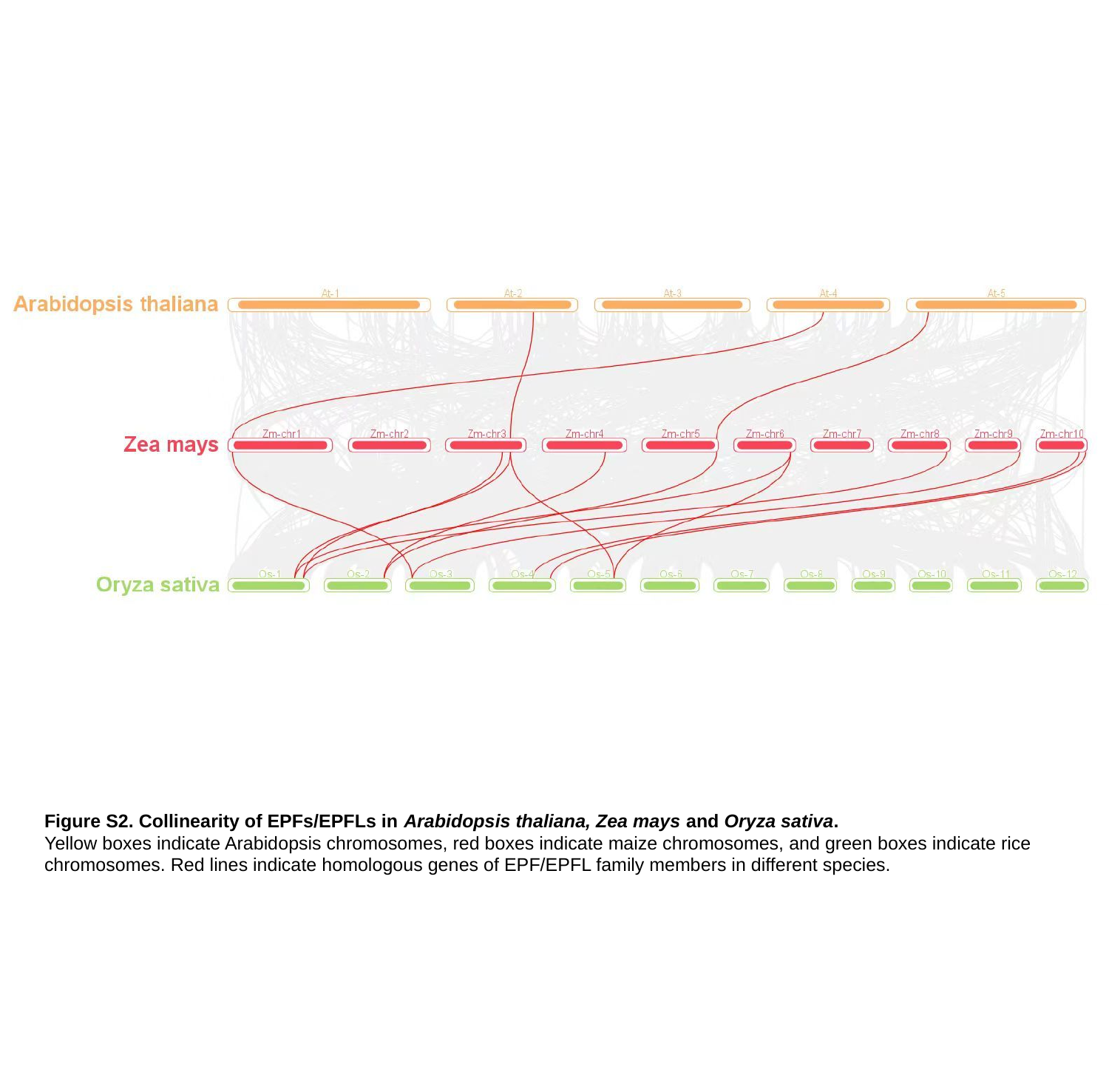

Figure S2. Collinearity of EPFs/EPFLs in Arabidopsis thaliana, Zea mays and Oryza sativa.
Yellow boxes indicate Arabidopsis chromosomes, red boxes indicate maize chromosomes, and green boxes indicate rice chromosomes. Red lines indicate homologous genes of EPF/EPFL family members in different species.

## Slide 3
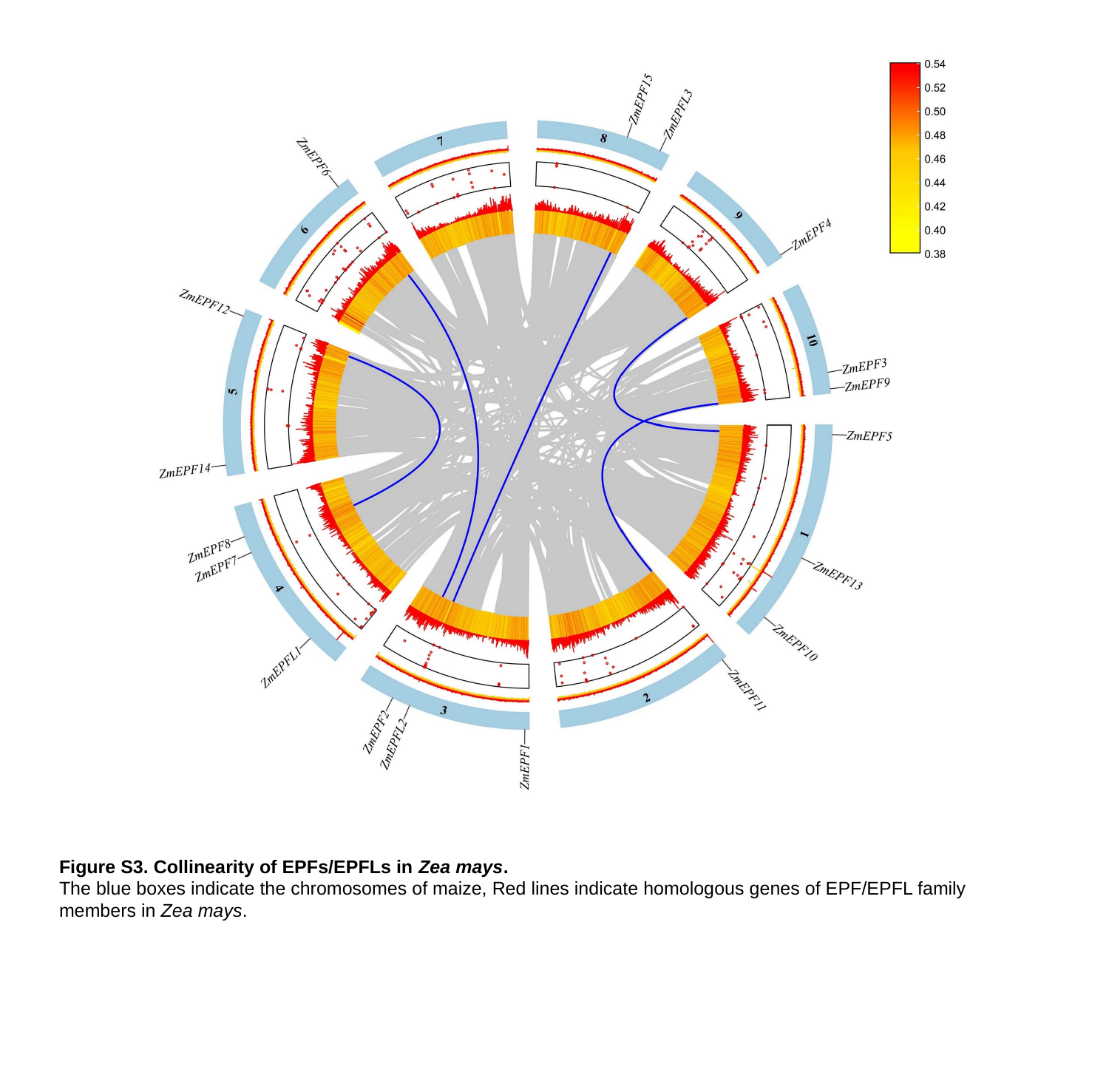

Figure S3. Collinearity of EPFs/EPFLs in Zea mays.
The blue boxes indicate the chromosomes of maize, Red lines indicate homologous genes of EPF/EPFL family members in Zea mays.
